# Evaluation of the Benefits, Satisfaction, and Limitations of Intergenerational Face-to-Face Activities: A General Population Survey in Spain

**DOI:** 10.3390/ijerph18189683

**Published:** 2021-09-14

**Authors:** Alejandro Canedo-García, Jesús-Nicasio García-Sánchez, Cristina Díaz-Prieto, Deilis-Ivonne Pacheco-Sanz

**Affiliations:** 1 Department of Psychology, Sociology and Philosophy, Universidad de León, 24071 León, Spain; acang@unileon.es (A.C.-G.); cdiap@unileon.es (C.D.-P.); 2 Department of Psychology, Universidad de Valladolid, 47011 Valladolid, Spain; deilisivonne.pacheco@uva.es

**Keywords:** intergenerational relationships, benefits, satisfaction, limitations, face-to-face activities

## Abstract

Over the last decades, social isolation and loneliness among older adults have given rise to an increased interest in the study of intergenerational relationships. Intergenerational programs provide a great opportunity for older adults to connect with people of other generations. Many studies have reported the beneficial effects of these programs, improving the mental and physical health of the elderly and contributing to better academic formation and social skills in children. The aim of this study was to examine the benefits, satisfaction, and limitations of the intergenerational interactions derived from the performance of face-to-face activities, such as daily and healthcare activities, educational, cultural and leisure activities, and physical or sport activities. Participants were subjects living in Spain of any age (grouped into three age groups: <22, 22–40, and >40 years old) and from different social groups who completed an electronic 88-item questionnaire. The analysis of sociodemographic variables of the survey respondents revealed that people aged 40 or more, with personal autonomy, single or married, and living with a partner and/or other relative, and not retired, performed face-to-face activities with people of other generations with a significantly higher frequency than the rest of the groups for each variable. Most participants who participated in intergenerational face-to-face activities reported benefits to their physical and mental health, mood, relationships, self-determination, social participation, and academic education. Most participants were quite or very satisfied with the person with whom they performed this type of activities, especially if this person was a friend or a close relative. Except for grandparents, people who participated in intergenerational face-to-face activities and who had no limitations or disabilities were more frequently reported by the participants. In conclusion, intergenerational interactions derived from the performance of face-to-face activities can contribute to improve both the physical and mental health, social skills, and relationships of all people involved.

## 1. Introduction

In recent decades, the worldwide population has aged as a result of declining fertility and mortality [1]. In 2019, the number of people aged 60 years and older was 1 billion and is expected to increase to 2.1 billion by 2050 [2]. At the same time, research shows an increase in loneliness and social isolation, which have a widely recognized negative impact on both mental and physical health [3]. In Spain, the percentage of old people reporting feelings of loneliness was 23.1%, and it contributes significantly to the explanation of mental health in this population [4]. Many researchers have recognized that interactions between people of different generations are key to reducing age discrimination [5] and the social isolation of older people [6].

Intergenerational programs can be defined as social activities to promote active aging among older adults through contact with other generations [7]. Previous studies have shown a wide spectrum of beneficial effects of these programs. In older populations, intergenerational programs improved physical and mental health, and increased social activities [8,9,10,11]. Children and young people also benefited from these programs through improvements in their academic formation, positive perception of the elderly, and attitudes toward community activities [12,13,14]. Moreover, studies show that creating intergenerational communities with both place-based and program-based support can contribute to increase social relationships across generations, thereby better addressing aging-related societal challenges [15].

In this context, we previously published a systematic review to identify the relevant elements that ensure the effectiveness of face-to-face and virtual intergenerational interventions [16]. We found that programs with a greater number of empirically based interventions controls had the greatest effectiveness, which was also modulated by other variables such as the participants’ disorder, their academic or literacy levels, membership of an organization, and situation of risk of exclusion. Moreover, we demonstrated an inverse relationship between the level of socio-communicative competence and the physical and mental condition of the subjects, and the number of empirically based criteria that programs meet.

We hypothesize that performing activities between people of different generations is essential to facilitate and improve the quality of life of older people, whether dependent or not, while promoting relationships and social values among young people and adults. Based on the evidence from studies that have described the health benefits of intergenerational programs in different populations, in the current work we wondered about the prevalence of intergenerational interactions and their derived benefits, satisfaction, and limitations in the Spanish population. Therefore, the aim of this study was to assess the benefits, satisfaction, and limitations of the intergenerational relationships derived from the performance of face-to-face activities, using a general population survey in subjects of all ages living in Spain.

## 2. Materials and Methods

### 2.1. Sample

Participants in this study were subjects living in Spain, randomly belong to the different Spanish autonomous communities, of any age, and from different social groups (place or origin, education, marital status, employment situation, income level, etc.) who completed an online questionnaire. Participants could not use the same IP address to avoid duplication and guarantee the participant identity. A total of 2013 individuals (608 men and 1405 women, 30.2% and 69.8%, respectively) completed the survey and were included in the final study. The mean age of the participants was 33.96 years (SD = 16.01) and ranged from 10 to 85 years old. The participants were also grouped into 3 age groups: <22, 22–40, and >40 years old, representing around 25%, 35%, and 40%, respectively, of the total size. The sociodemographic characteristics of the participants in the study are presented in results in Table 2.

### 2.2. Instrument

This study used the Acción Conjunta Intergeneracional (ACIG) face to face instrument (see Supplementary Materials) [17]. It consists of an online questionnaire that, through 6 scales and 14 sub-scales, analyzes the information provided by people of all ages in relation to the social support they perceive, the face-to-face intergenerational activities they perform with family, friends, acquaintances, or professionals, and a series of psychosocial variables to study the benefits derived from the performance of programs or activities between generations. The sociodemographic data of the participants (age, gender, place of origin, marital status, educational level, autonomy level, living arrangements, employment situation, and income level) were also collected by the questionnaire.

Face-to-face activities were grouped into (1) daily and healthcare activities; (2) educational, cultural, and leisure activities; and (3) physical and sport activities. For each group of activities, participants were asked the questions shown in Table 1. Responses were recategorized when required for the statistical analysis.

### 2.3. Procedure

Participants were recruited and completed the online questionnaire via the professional survey website Survey Monkey (Spain) during October 2017 [17]. The maximum time required to complete the questionnaire was 25–30 min. Once the questionnaires were completed, the data were extracted in Excel format and codified appropriately for statistical analyzes.

### 2.4. Data and Statistical Analysis

In the first place, descriptive and multivariate analyzes were carried out, using SPSS version 26 (IBM Corp., New York, NY, USA), to see the relationship between the different variables used. With this, the sample was refined, outliers were eliminated, and tables were drawn up from the demographic data and general information. Likewise, the reliability calculations were performed by internal consistency of the instrument (Cronbach’s alphas) and item-scale correlations, and if the items were eliminated, the selection of the items could thus be refined.

For the quantitative variables, a Shapiro–Wilk test was conducted to examine the normality of the data, and central position statistics (mean or median) and measures of dispersion (standard deviation, SD; or interquartile range, IQR) were calculated. Associations between the qualitative variables were assessed using a chi-square contingency table analysis. 

An exploratory factor analysis (EFA) was performed with half of the sample, to calculate the factors using the method of maximum likelihood and direct oblimin rotation, a recommended method when there is intercorrelation between the factors, as is the case. With this, it was possible to obtain the structure of the construct validity and the adequacy of the sample was checked using the Kaiser–Meyer–Olkin coefficients, which must be greater than 0.70; or Bartlett’s test of sphericity, with a probability less than 0.05, similar to the determinant probability. From the pattern matrix obtained, one can calculate the composite reliability (CR), which must be greater than 0.90; the average variance extracted (AVE) or convergent validity (CV), which must be greater than 0.50; as well as the square of the AVE to obtain the discriminant validity (DV), thereby being able to compare it with the inter-correlation matrix between the factors, which must be less than the discriminant validity. These calculations of the compound reliability or McDonald’s omega and convergent and discriminant validity were done by using spreadsheets in Excel. Furthermore, to describe the qualitative variables, we used frequencies and percentages. 

With the other half of the sample, the measurement model obtained with the EFA, for the confirmatory factor analysis (CFA), was calculated using AMOS version 26. In this case, to confirm the fit of the models, the common coefficients in this type of analysis, the TLI and CFI, were calculated, which must be greater than 0.90; as well as the RMSEA, with coefficients less than 0.08. For the implementation of the CFA from the EFA, Gaskination’s StatWiki plugin (http://statwiki.gaskination.com/index.php?title=Plugins, accessed on 21 May 2021) and the Pattern Matrix Model Builder from the pattern matrices were used, as well as the Model Fit Measures, the Validity and Reliability Test, and other functionalities of AMOS version 26.

When the participants did not complete all items in the questionnaire, missing values were resolved by removing pairs of data from the analysis. The significance level (α risk) was set at 5% (α = 0.05).

## 3. Results

### 3.1. Sociodemographic Analysis

The sociodemographic results of our survey are shown in Table 2. Among the 2013 respondents, 30.2% (*n* = 608) were male and 69.8% (*n* =1405) were female. The mean and median age of the participants was 33.96 and 26.00 years, respectively (SD = 16.01; min = 10 and max = 85 years).

Regarding performance of face-to-face activities with people of different generations, 21.4% (*n* = 431) of the respondents stated that they carried out daily and healthcare activities, 34.2% (*n* = 689) educational, cultural, and leisure activities, and 33.3% (*n* = 670) sport activities. We assessed whether there was any association between performing these types of activities and the sociodemographic variables of the participants in the survey. The results of the Pearson’s chi-square analysis are provided in Table 3.

We found that performing daily and healthcare activities with people of a different generation was associated with age, gender, marital status, living arrangement, employment situation, and income level (Table 3). Thus, the frequency of participants who reported performing this type of intergenerational face-to-face activities was significantly higher among female, single, or married people, and subjects living with a partner and/or other relatives, and a trend towards significance (*p* = 0.050) was observed among individuals with university studies. Conversely, participants under 22 years old, retired, and with an income level less than 1000 euros/month reported performing these activities less frequently than the rest of the groups for each variable.

Intergenerational interactions related to educational, cultural, and leisure activities were strongly associated with the age, autonomy level, marital status, living arrangements, employment situation, and income level of the participants in our survey (Table 3). When comparing the groups across each variable, participants aged 40 or more, with personal autonomy, single or married, living with a partner and/or other relatives, unemployed or employed, reported performing these activities with people of other generations at a significantly higher frequency, and tended to be significant (*p* = 0.059) for people living in an urban area vs. a rural area.

In relation to performance of physical or sport activities with people of other generation, associations with marital status and the employment situation of respondents were found, with people single or married, and not retired, reporting this more frequently (Table 3). In addition, female participants carried out this type of intergenerational activities more frequently than men.

### 3.2. Validation of the Instrument

#### 3.2.1. Reliability Due to Internal Consistency

The validation of the instrument was carried out by first calculating the reliability by internal consistency, obtaining Cronbach’s alphas higher than 0.90. For example, considering the joint activities: for Activities of Daily Living and Health Care (ADL), Training, Cultural and Leisure Activities (FORM), and Physical and Sports Activities (FyD) for the Perceived Benefit scales, a Cronbach’s alpha = 0.962 was found; for the Perceived Satisfaction scales, an alpha = 0.925 was found; and for the Perceived Limitations scales, an alpha = 0.947 was found.

#### 3.2.2. Exploratory Factor Analysis (EFA)

Second, through an exploratory factorial analysis (EFA), we confirmed the construct validity, the adequacy of the sample, the compound reliability, and the convergent and discriminant validity. For example, for the Perceived Benefits scales, three consistent factors are foreign, each relative to two fields of shared activities. The adequacy of the sample indicates a KMO and was above 0.81; the probability of the Bartlett test of sphericity was less than 0.001, as well as that of the determinant, 0.038. From the pattern matrices, the composite reliability (CR) or McDonald’s omega indices were obtained, which were above 0.90; the average variance extracted (AVE) was above 0.50, indicating adequate reliability and validity. For example, for the factors FyD, AVD, and FORM, respectively, CR = 0.894, 0.912, and 0.894; the AVE or CV = 0.54, 0.54, and 0.50, and considering the square of the AVE (DC), all the coefficients are greater than the intercorrelations between the factors, thus confirming their discriminant validity; respectively, CV = 0.73, 0.74, and 0.71, with the highest intercorrelation between the factors being 0.43. Thus, the composite reliability and convergent and discriminant validity are confirmed. Or, in the case of the Perceived Satisfaction scales, a sample adequacy index of KMO = 0.925 and Bartlett’s sphericity or determinant probabilities lower than 0.001 were obtained. For FyD, AVD, and FORM, respectively, the composite reliability (CR) values were 0.91, 0.88, and 0.92; the AVE or CV = 0.52, 0.50, and 0.51; and the square of the AVE, or DV, were 0.72, 0.71, and 0.71, which, when compared with the inter-correlations between the factors, is above 0.51, the highest of them. Therefore, the composite reliability and the convergent and discriminant validity are confirmed.

#### 3.2.3. Confirmatory Factor Analysis (CFA)

**Figure 1:**
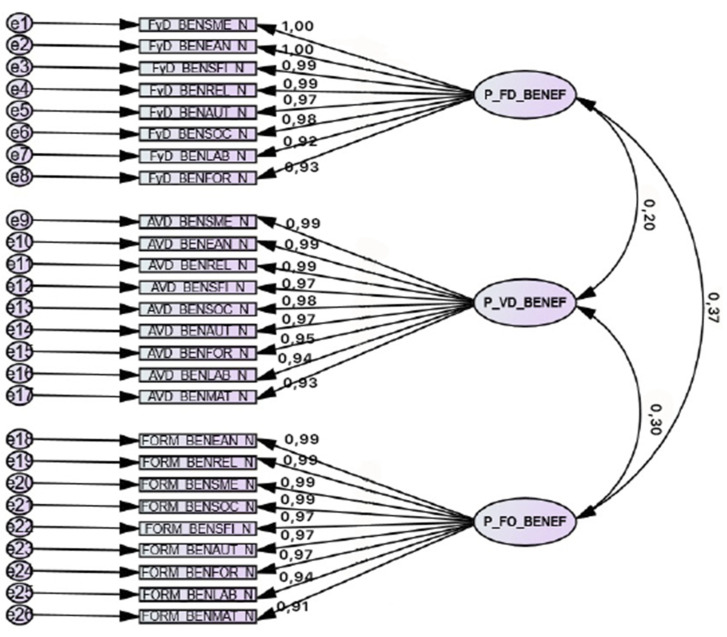
Confirmatory factor structure of the Perceived Benefit Scales (face-to-face). The latest proven model is presented. We get a TLI = 0.92, CFI = 0.93, and RMSEA = 0.075.

**Figure 2:**
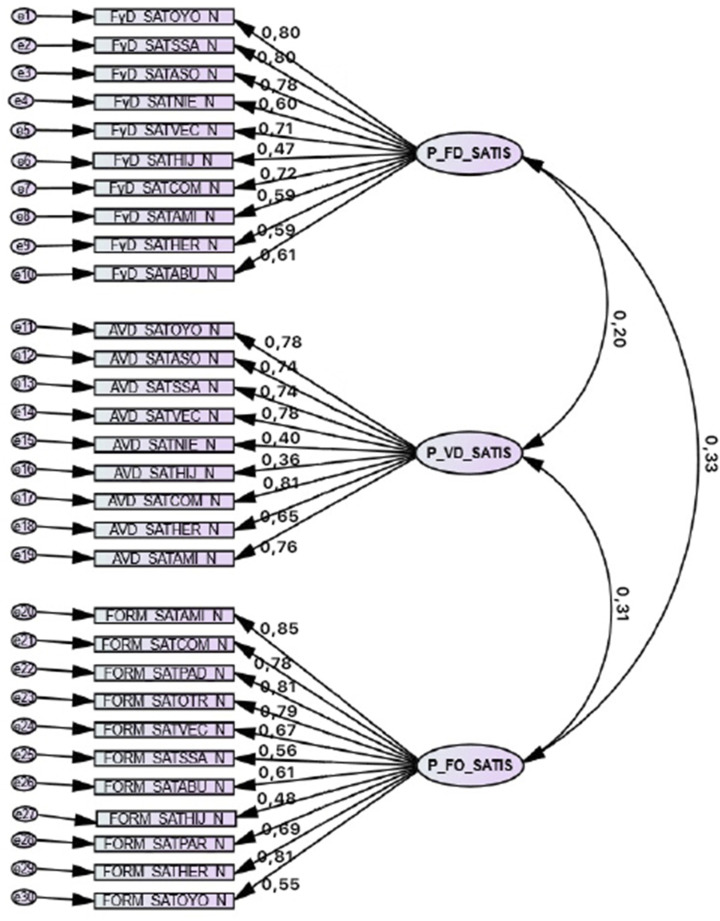
Confirmatory factor structure of the Perceived Satisfaction Scales (face-to-face). The latest proven model is presented. We get a TLI = 0.91, CFI = 0.92, and RMSEA = 0.078.

The structure resulting from the confirmatory factor analysis can be seen from the EFA pattern matrices, as shown in Figure 1 and Figure 2.

### 3.3. Benefits of Performing Intergenerational Face-to-Face Activities

Table 4 shows the benefits of performing face-to-face activities with people of another generation according to the respondents of our survey. Most participants agreed that performing daily and healthcare activities with people of another generation produces benefits for all the asked categories. High frequencies were found for the benefits to relationships (84.2%), mood (79.5%), mental health (76.9%), social participation (77.6%), self-determination (67.0%), and physical health (57.1%).

Regarding the performance of intergenerational activities related to education, culture, and leisure, the majority of the participants agreed that it was beneficial to their relationships (91.8%), mood (89.7%), social participation (88.5%), mental health (87.9%), academic education (75.6%), self-determination (71.7%), physical health (67.6%), and professional well- being (53.2%). Similar percentages of agreement were found among the respondents who performed physical or sport activities with someone of a different generation, benefitting their mood (89.3%), relationships (88.6%), mental health (87.3%), physical health (85.1%), social participation (77.4%), and self-determination (63.6%).

The characteristics age, gender, personal autonomy, and frequency of people with whom the participants in this study performed face-to-face activities are provided in Table 5 (age), Table 6 (gender), Table 7 (personal autonomy), and Table 8 (frequency).

### 3.4. Satisfaction of Performing Intergenerational Face-to-Face Activities

The participant in our survey were asked about the satisfaction they felt from performing face-to-face activities with people of another generation (Table 9). Considering daily and healthcare activities, most participants were quite or very satisfied with the person with whom they performed these activities. Interestingly, the greatest percentage was found when the person was a friend (88.45%), closely followed by the partner (87.3%), parent (86.3%), child (84.5%), grandparent (82.4%), other relative (80.7%), sibling (78.7%), colleague (75.4%), and a person in the same situation (72.7%) as the respondent. Similarly, participants were quite or very satisfied carrying out educational, cultural, and leisure activities with a friend (95.7%), partner (94.3%), parent (92.3%), sibling (91.1%), child (90.7%), grandparent (90.3%), other relative (89.9%), colleague (86.5%), and a person in their same situation (80.8%). In relation to physical or sport activities, people with whom the participants were quite or very satisfied more frequently were a friend (93.4%), partner (92.1%), sibling (91.4%), other relative (88.3%), colleague (85.9%), child (84.9%), grandparent (84.3%), and neighbor (83.1%).

### 3.5. Limitations of People Who Perform Intergenerational Face-to-Face Activities

An important issue that can condition the performance of intergenerational face-to-face activities is the possible limitations or disabilities of the people involved. Thus, we asked the participants in this study if the people with whom they performed these activities had any limitation. Limitations include visual, hearing, psychic, motor, learning, behavioral, communicational, and other disabilities, as well as autism spectrum and attention deficit disorders. The majority of the participants reported that the person with whom they performed the face-to-face activities had no limitations or disabilities (Table 10). Among the people who had any limitation, they were more frequently a grandparent, with a frequency of 53.7%, 43.8%, and 42.1% for daily and healthcare activities, educational activities, and sport activities, respectively.

## 4. Discussion

As a result of the increased distance, at the level of relationship and interaction, between people of different generations present in current societies, there is a growing interest in the study of intergenerational interactions and the benefits of programs that encourage the performance of intergenerational activities, in both face-to-face and virtual modalities. In this study, we conducted an online survey to evaluate the benefits, the satisfaction, and the limitations derived from performing face-to-face activities with people of different generations. Participants included were people living in Spain of any age and belonging to different social groups.

We firstly analyzed the sociodemographic characteristics of the respondents in order to test the associations with their participation in intergenerational face-to-face activities. We found that performing daily and healthcare activities with people of a different generation was associated with age, marital status, living arrangement, employment situation, and income level. Intergenerational interactions related to education, culture, and leisure were strongly associated with the age, autonomy level, marital status, living arrangements, employment situation, and income level. In relation to physical or sport activities, associations with the marital status and the employment situation were observed. These results suggest that it is important that intergenerational programs focus on promoting face-to-face activities, especially among people belonging to social groups that reported less intergenerational relationships, such as young people, widowers, divorcees, retirees, people who live alone, those with poor personal autonomy, or those with a low level of income.

Other researchers have found similar results in relation to the age of people that participate in intergenerational programs. Murayama et al. [18] conducted a study based on The Research of Productivity by Intergenerational Sympathy (REPRINTS) program in Japan, which trains volunteers over the age of 60 to read picture books to school children. They found that people between 30 and 59 years of age and people over 60 years of age have more positive effects on neighborhood trust than do people between 20 and 39 years of age [18]. Li et al. [19] examined the role of geographical proximity between intergenerational solidarity and life satisfaction. They found that the frequency of in-person contact was reduced when the geographical proximity between the parent and child increases. In addition, gender differences existed in the relationships between generations. The in-person contact with children played an important role in the life satisfaction of older men in empty-nest families, while the affectual and functional aspects of the intergenerational solidarity promoted life satisfaction for older women [19]. Other authors have also reported that older women receiving higher emotional and instrumental support from children have better health and well-being compared to older men [20,21,22].

According to a recent review [5], interventions that include both educational and intergenerational contact components show particularly strong effects, especially for combating negative attitudes toward aging. These findings suggest that interventions that promote intergenerational relationships are essential to combat age discrimination and also improve the health and well-being of older people. In addition, many previous studies have shown that the participation in intergenerational programs produces benefits for all the people involved [23].

In the elderly, the reported benefits of intergenerational programs include improvements in their physical and psychological health, cognitive function, well-being, physical activity, and social relationships. Regarding physical health, researchers have found that the performance of intergenerational programs were positively associated with the functional capacity of older adults [24,25,26], and a significant reduction in functional limitations of older people who participated in these programs [27,28]. In terms of improvements in psychological health, studies reported the positive influences of intergenerational interactions on generativity in aging [29,30], and associations with reduced depression among older populations [27]. Murayama et al. [10] found that the participation in the intervention research project REPRINTS was associated with a sense of manageability, which was also significantly related to depressive mood.

Regarding the effects of intergenerational interactions on the cognitive function of older people, Carlson et al. demonstrated that a community-based intergenerational program had a beneficial impact on promoting executive function, memory, and brain activity [9,31]. Similarly, the REPRINTS intergenerational program had long-term, positive effects that help maintain and promote the intellectual activity and physical functioning of participants [11]. In relation to the well-being and quality of life of older adults, Chippendale and Boltz [9,32] investigated the effects of an intervention in which community-dwelling older adults shared their knowledge and life experiences with health science students, and found that this program enhanced the participants’ sense of purpose and meaning in life, a factor known to prevent cognitive loss and disability in older people. Park et al. [33] studied the health benefits of Program 60, a university-based lifelong learning program, in adults aged 60 and older, and observed an increased emotional satisfaction and quality of life of participants.

Intergenerational interactions often offer a great opportunity to reduce sedentary lifestyle and to promote healthy physical activities, such as walking. Several studies revealed that intergenerational interactions were positively related to the total physical activity of older people [8,34,35]. Varma [36] reported that an intergenerational volunteering intervention increased the walking activity among older women but had no effect on walking among older men. In addition, other studies have shown that older people who regularly participate in activities with children and youth experienced fewer falls, relied less on a cane [37,38,39], and demonstrated an increase in social activities, such as visiting friends, reading, watching TV, etc.

Interactions between different generations also have recognized benefits for children and young people. Studies have provided evidence that intergenerational programs promote positive changes, such as a reduction in the aging-related stereotypes that are common in this group of age [12,13,40], and an improvement in their self-confidence and feelings about social responsibility [41,42]. A study conducted by Yasunaga et al. [43] showed that the students who participated in an intergenerational picture book reading program were relieved from the mental and physical stress response. Regarding the academic field, many researchers have found that intergenerational exchange improved school engagement and decreased school absenteeism [44]. Moreover, children and adolescents can also benefit from intergenerational programs by providing access to adults at difficult times, reducing involvement in offending behavior and drug use, and improving personal resilience [45].

In our study, a high number of participants reported that performing intergenerational face-to-face activities produces benefits on their physical and mental health, mood, relationships, self-determination, social participation, and academic education. In addition, most participants were quite or very satisfied with the person with whom they perform these type of activities, especially if this person is a friend or a close relative, such as their partner, a parent, a grandparent, a child, or a sibling. Finally, the people who participated in intergenerational face-to-face activities and who had no limitations or disabilities were more frequently reported by the respondents of the survey, except when the person was their grandparent.

Nonetheless, this study presents a series of limitations that must be taken into consideration. First, the sample bias could be present since voluntary sampling was used. As a result, factors such as motivation to complete the questionnaire, the availability of technological resources, or the level of digital competence of the participants may have influenced the final sample obtained. Furthermore, these aspects may also have conditioned the sociodemographic, educational, and economic characteristics of the study sample, reducing the generalizability of our findings. In addition, subjects who were not independent in their activities of daily living were not included in the study due to their lack of ability to complete the online survey. Despite these limitations, our study presents findings that can contribute to support the benefits of the intergenerational interactions derived from face-to-face activities.

Likewise, the assessment of the benefits, satisfaction, and limitations of intergenerational face-to-face activities can be further amplified given the context of the COVID-19 pandemic and post-pandemic.

## 5. Conclusions

The results suggest that intergenerational interactions derived from performing face-to-face activities, such as daily and healthcare activities, educational, cultural, and leisure activities, and physical or sport activities, can contribute to improve both the physical and mental health, social skills, and relationships of all people involved in them. Additionally, these type of interactions may improve the satisfaction of people of different generations and reduce the physical and cognitive limitations of the subjects who participate in intergenerational face-to-face activities.

## Figures and Tables

**Table 1:** Original coding and recoding of the possible answers of participants carrying out activities (1), (2), or (3) on several categories: (A) benefit; (B) people with whom and frequency; (C) age; (D) gender; (E) autonomy; (F) limitations; and (G) satisfaction with whom they carry out those activities.

(A) Performing activities with people of another generation produces BENEFITS for your: -Physical health, -Mental health, -Mood, -Relationships, -Self-determination, -Social participation, -Economic well-being, -Professional well-being, -Academic education. RC: Totally disagree—Disagree—Rather disagree—Neither agree nor disagree—Rather agree—Agree—Totally agree. RR: Disagree—Neither agree nor disagree—Agree.
(B) With WHO & FREQUENCY do you perform activities? -Partner, -Child, -Grandchild, -Parent, -Grandparent, -Sibling, -Other relative, -Friend, -Neighbor, -Colleague, -Person in the same situation, -Professional of an institution, -Professional of health, social or academic services. RC: Sometime a year—Sometime a month—Sometime a week—Every day or almost every day. RR: Sometime a year/month—Sometime a week/Every day or almost every day.
(C) AGE of the people with whom you perform activities: -Partner, -Child, -Grandchild, -Parent, -Grandparent, -Sibling, -Other relative, -Friend, -Neighbor, -Colleague, -Person in the same situation, -Professional of an institution, -Professional of health, social or academic services. RC: <6–6/14–15/20–21/39–40/59–60/65–66/70–71–75–>75 years old. RR: 0/14–15/65–>65 years old.
(D) GENDER of the people with whom you perform activities: -Partner, -Child, -Grandchild, -Parent, -Grandparent, -Sibling, -Other relative, -Friend, -Neighbor, -Colleague, -Person in the same situation, -Professional of an institution, -Professional of health, social or academic services. RC & RR: Male—Female
(E) AUTONOMY of the people with whom you perform activities: -Partner, -Child, -Grandchild, -Parent, -Grandparent, -Sibling, -Other relative, -Friend, -Neighbor, -Colleague, -Person in the same situation, -Professional of an institution, -Professional of health, social or academic services. RC: Not need support—Need family—Need professional—Need other support. RR: Not need support—Need support.
(F) LIMITATION of the people with whom you perform activities: -Partner,- Child, -Grandchild, -Parent, -Grandparent, -Sibling, -Other relative, -Friend, -Neighbor, -Colleague, -Person in the same situation, -Professional of an institution, -Professional of health, social or academic services. RC: Without disability—Visual disability—Hearing disability—Psychic disability—Motor disability—Learning disability—Behavioral disability—Communication disability—Autism spectrum disorders—Attention deficit disorders—Others. RR: No limitation—Any limitation.
(G) SATISFACTION you feel from perform activities with these people: -Partner, -Child, -Grandchild, -Parent, -Grandparent, -Sibling, -Other relative, -Friend, -Neighbor, -Colleague, -Person in the same situation, -Professional of an institution, -Professional of health, social or academic services. RC: Not satisfied at all—Little– Somewhat—Quite—Very satisfied. RR: Not/little satisfied—Neither satisfied nor dissatisfied—Quite/very satisfied.

Note: (1) = daily/healthcare activities; (2) = educational/cultural/leisure activities; (3) = physical/sport activities. RC = response categories; RR = recategorization of responses.

**Table 2:** Sociodemographic characteristics of the participants in the study.

Variables	*N* (%)
Age (years)	33.96 ^1^ (16.01) ^2^
Gender	
Male	608 (30.2)
Female	1405 (69.8)
Place of origin	
Rural area, small village	440 (21.9)
Rural area, large village	326 (16.2)
Urban area, small town	906 (45.0)
Urban area, large town	341 (16.9)
Education	
Primary school	20 (1.0)
High school	210 (10.4)
Vocational training	138 (6.9)
College or university	1645 (81.7)
Autonomy level	
Alone	1705 (84.7)
Family support	253 (12.6)
Professional support	12 (0.6)
Other support	43 (2.1)
Marital status	
Single	1024 (50.9)
Married or in union	761 (37.8)
Widowed	23 (1.1)
Separated	25 (1.2)
Divorced	56 (2.8)
Living arrangements	
Living alone	205 (10.2)
Living with a partner	332 (16.5)
Living with a partner and children	342 (17.0)
Living with a partner and grandchildren	3 (0.1)
Living with a partner, children and grandchildren	5 (0.2)
Living with children	36 (1.8)
Living with children and grandchildren	3 (0.1)
Living with parents	562 (27.9)
Living with grandparents	11 (0.5)
Living with parents and grandparents	43 (2.1)
Living with other relatives	43 (2.1)
Living with friends	248 (12.3)
Other types	180 (8.9)
Employment situation	
Unemployed	938 (46.6)
Employed	913 (45.4)
Retired	151 (7.5)
Income level (EUR/month)	
>2500	862 (42.8)
2001–2500	213 (10.6)
1501–2000	264 (13.1)
1001–1500	229 (11.4)
501–1000	204 (10.1)
<500	116 (5.8)

Note: *N* = number of participants; % = percentage; ^1^ mean; ^2^ standard deviation.

**Table 3:** Association between performing daily/healthcare, educational/cultural/leisure, and physical/sport face-to-face activities with people of different generations and the sociodemographic characteristics of the participants.

Variables	Daily/Healthcare Activities			Educational/Cultural/Leisure Activities			Physical/Sport Activities		
	*N* (%)	χ^2^	*p*	*N* (%)	χ^2^	*P*	*N* (%)	χ^2^	*p*
Age (years)									
<22 22–40 ≥40	105 (24.5) 158 (36.8) 166 (38.8)	9.98	0.007	158 (23.0) 229 (33.4) 299 (43.6)	38.25	<0.001	164 (24.5) 257 (38.4) 249 (37.2)	4.34	0.114
Gender									
Male Female	108 (25.1) 323 (74.9)	10.44	0.001	204 (29.6) 485 (70.4)	1.83	0.177	230 (34.3) 440 (65.7)	3.55	0.600
Place of origin									
Rural area Urban area	157 (36.4) 274 (63.6)	0.94	0.332	240 (34.8) 449 (65.2)	3.56	0.059	263 (39.3) 407 (60.7)	2.10	0.147
Education									
Less than college or university College or university	65 (15.1) 366 (84.9)	3.84	0.050	112 (16.3) 577 (83.7)	1.35	0.245	122 (18.2) 548 (81.8)	1.07	0.300
Autonomy level									
Alone Family/professional/other support	362 (84.0) 69 (16.0)	1.35	0.246	571 (82.9) 118 (17.1)	8.44	0.004	577 (86.1) 93 (13.9)	0.01	0.909
Marital status									
Single Married or in union Widowed/separated/divorced	193 (47.9) 196 (48.6) 14 (3.5)	15.63	<0.001	291 (45.0) 300 (46.4) 56 (8.7)	31.47	<0.001	320 (50.9) 265 (42.1) 44 (7.0)	6.11	0.047
Living arrangements									
Living alone/with children/with grandchildren Living with a partner/a partner and children and/or grandchildren Living with parents and/or grandparents/ other relatives Living with friends/other types	41 (9.7) 170 (39.4) 158 (36.7) 61 (14.2)	25.21	<0.001	98 (14.2) 280 (40.6) 197 (28.6) 114 (16.5)	19.63	<0.001	84 (12.5) 248 (37.0) 200 (29.9) 138 (20.6)	0.84	0.839
Employment situation									
Unemployed Employed Retired	180 (41.8) 229 (53.1) 22 (5.1)	23.02	<.001	268 (38.9) 330 (47.9) 91 (13.2)	44.72	<0.001	310 (46.3) 297 (44.3) 63 (9.4)	7.08	0.029
Income level (€/month)									
>2001 1001–2000 <1000	230 (53.4) 126 (29.2) 75 (17.4)	7.19	0.028	348 (50.5) 193 (28.0) 171 (25.5)	22.31	<0.001	374 (55.8) 148 (21.5) 125 (18.7)	1.93	0.380

Note: *N* = number of participants; χ^2^ = chi-square test; *p* = significance; α-Risk = 0.05.

**Table 4:** Benefits reported by participants who performed intergenerational face-to-face activities.

	Daily/Healthcare Activities *N* (%)			Educational/Cultural/Leisure Activities *N* (%)			Physical/Sport Activities *N* (%)		
	Disagr ee	NA/ND	Agree	Disagr ee	NA/ND	Agree	Disagr ee	NA/ND	Agree
Physic al health	52	78	173	42	124	347	20	61	463
	(17.2)	(25.7)	(57.1)	(8.2)	(24.2)	(67.6)	(3.7)	(11.2)	(85.1)
Mental health	33	37	233	21	41	451	18	51	475
	(10.9)	(12.2)	(76.9)	(4.1)	(8.0)	(87.9)	(3.3)	(9.4)	(87.3)
Mood	33	29	241	19	34	460	17	41	486
	(10.9)	(9.6)	(79.5)	(3.7)	(6.6)	(89.7)	(3.1)	(7.5)	(89.3)
Relationship s	23	25	255	14	28	471	18	44	482
	(7.6)	(8.3)	(84.2)	(2.7)	(5.5)	(91.8)	(3.3)	(8.1)	(88.6)
Self-determ ination	29	71	203	35	110	368	35	163	346
	(9.6)	(23.4)	(67.0)	(6.8)	(21.4)	(71.7)	(6.4)	(30.0)	(63.6)
Social partici pation	19	49	235	21	38	454	23	100	421
	(6.3)	(16.2)	(77.6)	(4.1)	(7.4)	(88.5)	(4.2)	(18.4)	(77.4)
Economic well-being	77	103	123	136	186	191	145	236	163
	(25.4)	(34.0)	(40.6)	(26.5)	(36.3)	(37.2)	(26.7)	(43.4)	(30.0)
Professional well-being	68	110	125	80	160	273	118	217	209
	(22.4)	(36.3)	(41.3)	(15.6)	(31.2)	(53.2)	(21.7)	(39.9)	(38.4)
Acade mic educat ion	50	97	156	43	82	388	95	189	260
	(16.5)	(32.0)	(51.5)	(8.4)	(16.0)	(75.6)	(17.5)	(34.7)	(47.8)

Note: *N* = number of participants; % = percentage; NA/ND = neither agree nor disagree.

**Table 5:** Age of people with whom the participants performed intergenerational face-to-face activities.

	Daily/Healthcare Activities *N* (%)			Educational/Cultural/Leisure Activities *N* (%)			Physical/Sport Activities *N* (%)		
	0–14	15–65	>65	0–14	15–65	>65	0–14	15–65	>65
Partner	2	172	7	5	292	23	5	295	11
	(1.1)	(95.0)	(3.9)	(1.6)	(91.3)	(7.2)	(1.6)	(94.9)	(3.5)
Child	66	30	1	89	86	1	92	74	0
	(68.0)	(30.9)	(1.0)	(50.6)	(48.9)	(0.6)		(44.6)	(0)
Grandchild	13	1	1	43	6	2	48	6	2
	(86.7)	(6.7)	(6.7)	(84.3)	(11.8)	(3.9)	(85.7)	(10.7)	(3.6)
Parent	1	137	96	3	207	105	5	218	68
	(0.4)	(58.5)	(41.0)	(1.0)	(65.7)	(33.3)	(1.7)	(74.9)	(23.4)
Grandparent	0	4	112	3	10	130	4	6	110
	(0)	(3.4)	(96.6)	(2.1)	(7.0)	(90.9)	(3.3)	(5.0)	(91.7)
Sibling	15	144	3	15	240	5	19	218	3
	(9.3)	(88.9)	(1.9)	(5.8)	(92.3)	(1.9)	(7.9)	(90.8)	(1.3)
Other relative	9	87	27	15	168	20	27	160	14
	(7.3)	(70.7)	(22.0)	(7.4)	(82.8)	(9.9)	(13.4)	(79.6)	(7.0)
Friend	1	151	5	4	359	24	6	359	17
	(0.6)	(96.2)	(3.2)	(1.0)	(92.8)	(6.2)	(1.6)	(94.0)	(4.5)
Neighbor	2	61	14	10	135	22	9	157	12
	(2.6)	(79.2)	(18.2)	(6.0)	(80.8)	(13.2)	(5.1)	(88.2)	(6.7)
Colleague	3	104	0	4	244	9	6	180	6
	(2.8)	(97.2)	(0)	(1.6)	(94.9)	(3.5)	(3.1)	(93.8)	(3.1)
Person in the same situation	3	50	0	4	93	6	10	74	4
	(5.7)	(94.3)	(0)	(3.9)	(90.3)	(5.8)	(11.4)	(84.1)	(4.5)
Professional of an institution	4	43	2	6	96	10	12	77	6
	(8.2)	(87.8)	(4.1)	(5.4)	(85.7)	(8.9)	(12.6)	(81.1)	(6.3)
Professional of social services	4	42	0	4	88	4	11	59	6
	(8.7)	(91.3)	(0)	(4.2)	(91.7)	(4.2)	(14.5)	(77.6)	(7.9)

Note: *N* = number of participants; % = percentage.

**Table 6:** Gender of people with whom the participants performed intergenerational face-to-face activities.

	Daily/Healthcare Activities *N* (%)		Educational/Cultural/Leisure Activities *N* (%)		Physical/Sport Activities *N* (%)	
	Male	Female	Male	Female	Male	Female
Partner	130 (73.9)	46 (26.1)	217 (69.3)	96 (30.7)	205 (66.6)	103 (33.4)
Child	47 (50.0)	47 (50.0)	91 (53.2)	80 (46.8)	96 (59.3)	66 (40.7)
Grandchild	10 (66.7)	5 (33.3)	23 (52.3)	21 (47.7)	34 (58.6)	24 (41.4)
Parent	52 (23.0)	174 (77.0)	86 (29.2)	209 (70.8)	102 (36.7)	176 (63.3)
Grandparent	21 (18.3)	94 (81.7)	31 (23.7)	100 (76.3)	41 (35.0)	76 (65.0)
Sibling	77 (48.1)	83 (51.9)	121 (47.8)	132 (52.2)	123 (51.5)	116 (48.5)
Other relative	55 (45.1)	67 (54.9)	77 (37.7)	127 (62.3)	86 (43.9)	110 (56.1)
Friend	50 (33.1)	101 (66.9)	134 (36.2)	236 (63.8)	178 (47.6)	196 (52.4)
Neighbor	36 (48.6)	38 (51.4)	69 (42.9)	92 (57.1)	91 (51.7)	85 (48.3)
Colleague	42 (40.8)	61 (59.2)	99 (41.1)	142 (58.9)	99 (51.8)	92 (48.2)
Person in the same situation	18 (35.3)	33 (64.7)	35 (36.5)	61 (63.5)	32 (37.6)	53 (62.4)
Professional of an institution	22 (44.0)	28 (56.0)	48 (44.4)	60 (55.6)	48 (53.9)	41 (46.1)
Professional of social services	23 (46.9)	26 (53.1)	50 (53.8)	43 (46.2)	45 (58.4)	32 (41.6)

Note: *N* = number of participants; % = percentage.

**Table 7:** Autonomy level of people with whom the participants performed intergenerational face-to-face activities.

	Daily/Healthcare Activities *N* (%)		Educational/Cultural/Leisure Activities *N* (%)		Physical/Sport Activities *N* (%)	
	Not NS	NS	Not NS	NS	Not NS	NS
Partner	171 (92.4)	14 (7.6)	302 (95.3)	15 (4.7)	302 (97.1)	9 (2.9)
Child	63 (64.3)	35 (35.7)	148 (81.8)	33 (18.2)	152 (91.0)	15 (9.0)
Grandchild	16 (84.2)	3 (15.8)	44 (84.6)	8 (15.4)	53 (88.3)	7 (11.7)
Parent	160 (66.9)	79 (33.1)	271 (85.5)	46 (14.5)	266 (90.8)	27 (9.2)
Grandparent	32 (26.2)	90 (73.8)	74 (49.7)	75 (50.3)	75 (58.6)	53 (41.4)
Sibling	128 (78.5)	35 (21.5)	232 (89.9)	26 (10.1)	235 (95.5)	11 (4.5)
Other relative	94 (75.2)	31 (24.8)	188 (87.0)	28 (13.0)	192 (90.6)	20 (9.4)
Friend	124 (84.4)	23 (15.6)	348 (93.5)	24 (6.5)	370 (96.1)	15 (3.9)
Neighbor	61 (81.3)	14 (18.7)	151 (86.8)	23 (13.2)	174 (95.1)	9 (4.9)
Colleague	89 (89.0)	11 (11.0)	235 (93.3)	17 (6.7)	190 (95.5)	9 (4.5)
Person in the same situation	41 (73.2)	15 (26.8)	89 (86.4)	14 (13.6)	92 (93.9)	6 (6.1)
Professional of an institution	42 (77.8)	12 (22.2)	101 (92.7)	8 (7.3)	92 (97.9)	2 (2.1)
Professional of social services	42 (87.5)	6 (12.5)	94 (94.9)	5 (5.1)	79 (97.5)	2 (2.5)

Note: *N* = number of participants; % = percentage; NS = need support.

**Table 8:** People with whom the participants performed intergenerational face-to-face activities and their frequency.

	Daily/Healthcare Activities *N* (%)		Educational/Cultural/Leisure Activities *N* (%)		Physical/Sport Activities *N* (%)	
	Sometime a Year/Month	Sometime a Week/(Almost) Everyday	Sometime a Year/Month	Sometime a Week/(Almost) Everyday	Sometime a Year/Month	Sometime a Week/(Almost) Everyday
Partner	36 (20.6)	139 (79.4)	98 (31.3)	215 (68.7)	137 (43.6)	177 (56.4)
Child	28 (27.5)	74 (72.5)	76 (41.5)	107 (58.5)	101 (59.4)	69 (40.6)
Grandchild	17 (77.3)	5 (22.7)	34 (59.6)	23 (40.4)	47 (77.0)	14 (23.0)
Parent	55 (24.3)	171 (75.7)	171 (54.5)	143 (45.5)	170 (58.0)	123 (42.0)
Grandparent	48 (41.4)	68 (58.6)	91 (62.8)	54 (37.2)	94 (77.0)	28 (23.0)
Sibling	82 (51.3)	78 (48.8)	161 (61.5)	101 (38.5)	164 (67.5)	79 (32.5)
Other relative	77 (59.7)	52 (40.3)	159 (72.3)	61 (27.7)	151 (75.5)	49 (24.5)
Friend	74 (44.6)	92 (55.4)	147 (37.4)	246 (62.6)	153 (38.7)	242 (61.3)
Neighbor	76 (85.4)	13 (14.6)	117 (63.2)	68 (36.8)	112 (62.2)	68 (37.8)
Colleague	57 (51.8)	53 (48.2)	161 (59.0)	112 (41.0)	121 (59.0)	84 (41.0)
Person in the same situation	43 (64.2)	24 (35.8)	81 (68.6)	37 (31.4)	79 (80.6)	19 (19.4)
Professional of an institution	48 (76.2)	15 (23.8)	84 (62.7)	50 (37.3)	79 (75.2)	26 (24.8)
Professional of social services	49 (72.1)	19 (27.9)	86 (73.5)	31 (26.5)	69 (77.5)	20 (22.5)

Note: *N* = number of participants; % = percentage.

**Table 9:** Level of satisfaction reported by participants who performed intergenerational face-to-face activities.

	Daily/Healthcare Activities *N* (%)			Educational/Cultural/Leisure Activities *N* (%)			Physical/Sport Activities *N* (%)		
	Not/Little Satisfied	NS/ ND	Quite/Very Satisfied	Not/Little Satisfied	NS/ ND	Quite/Very Satisfied	Not/Little Satisfied	NS/ ND	Quite/Very Satisfied
Partner	13 (6.6)	12 (6.1)	172 (87.3)	10 (3.2)	8 (2.5)	296 (94.3)	8 (2.6)	16 (5.1)	287 (92.3)
Child	12 (10.3)	6 (5.2)	98 (84.5)	14 (7.7)	3 (1.6)	165 (90.7)	15 (9.0)	10 (6.0)	141 (84.9)
Grandchild	8 (32.0)	2 (8.0)	15 (60.0)	12 (21.8)	3 (2.5)	40 (72.7)	15 (23.8)	5 (7.9)	43 (68.3)
Parent	13 (5.1)	22 (8.6)	221 (86.3)	9 (2.9)	15 (4.8)	288 (92.3)	5 (1.7)	18 (6.2)	268 (92.1)
Grandparent	6 (4,6)	17 (13.0)	108 (82.4)	6 (4.1)	8 (5.5)	131 (90.3)	6 (5.0)	13 (10.7)	102 (84.3)
Sibling	12 (7.1)	24 (14.2)	133 (78.7)	8 (3.1)	15 (5.8)	235 (91.1)	9 (3.7)	12 (4.9)	222 (91.4)
Other relative	5 (3.6)	22 (15.7)	113 (80.7)	8 (3.5)	15 (6.6)	204 (89.9)	7 (3.4)	17 (8.3)	182 (88.3)
Partner	5 (3.0)	14 (8.5)	145 (88.4)	8 (2.1)	8 (2.1)	360 (95.7)	7 (1.8)	18 (4.7)	356 (93.4)
Neighbor	9 (11.1)	21 (25.9)	51 (63.0)	10 (5.8)	34 (19.8)	128 (74.4)	9 (4.9)	22 (12.0)	152 (83.1)
Colleague	6 (5.3)	22 (19.3)	86 (75.4)	9 (3.7)	24 (9.8)	212 (86.5)	9 (4.7)	18 (9.4)	165 (85.9)
Person in the same situation	9 (16.4)	6 (10.9)	40 (72.7)	10 (10.1)	9 (9,1)	80 (80.8)	13 (14.9)	14 (16.1)	60 (69.0)
Professional of an institution	7 (12.7)	13 (23.6)	35 (63.3)	14 (12.1)	13 (11.2)	89 (76.7)	14 (15.1)	10 (10.8)	69 (74.2)
Professional of Social services	9 (15.5)	10 (17.2)	39 (67.2)	11 (11.0)	14 (14.0)	75 (75.0)	14 (17.5)	11 (13.8)	55 (68.8)

Note: *N* = number of participants; % = percentage; NS/ND = neither satisfied nor dissatisfied.

**Table 10:** Limitations of people with whom participants performed intergenerational face-to-face activities.

	Daily/Healthcare Activities *N* (%)		Educational/Cultural/Leisure Activities *N* (%)		Physical/Sport Activities *N* (%)	
	No Limitation	Any Limitation	No Limitation	Any Limitation	No Limitation	Any Limitation
Partner	146 (89.6)	17 (10.4)	272 (88.6)	35 (11.4)	276 (90.5)	29 (9.5)
Child	64 (68.8)	29 (31.2)	146 (83.0)	30 (17.0)	143 (86.7)	22 (13.3)
Grandchild	16 (76.2)	5 (23.8)	42 (79.2)	11 (20.8)	56 (86.2)	9 (13.8)
Parent	142 (63.1)	83 (36.9)	245 (79.8)	62 (20.2)	246 (84.2)	46 (15.8)
Grandparent	56 (46.3)	65 (53.7)	81 (56.3)	63 (43.8)	77 (57.9)	56 (42.1)
Sibling	126 (83.4)	25 (16.6)	226 (91.5)	21 (8.5)	217 (90.8)	22 (9.2)
Other relative	82 (72.6)	31 (27.4)	170 (82.1)	37 (17.9)	178 (87.7)	25 (12.3)
Friend	120 (85.1)	21 (14.9)	318 (90.1)	35 (9.9)	342 (90.7)	35 (9.3)
Neighbor	62 (81.6)	14 (18.4)	141 (83.4)	28 (16.6)	164 (89.1)	20 (10.9)
Colleague	81 (89.0)	10 (11.0)	208 (88.1)	28 (11.9)	173 (91.1)	17 (8.9)
Person in the same situation	44 (77.2)	13 (22.8)	83 (79.8)	21 (20.2)	76 (83.5)	15 (16.5)
Professional of an Institution	40 (78.4)	11 (21.6)	92 (80.2)	23 (20.0)	83 (83.8)	16 (16.2)
Professional of Social services	38 (82.6)	8 (17.4)	84 (84.0)	16 (16.0)	67 (84.8)	12 (15.2)

Note: *N* = number of participants; % = percentage.

## Data Availability

The datasets generated for this study are available on request to the corresponding author.

## Supplementary Materials

The following are available online at https://www.mdpi.com/article/10.3390/ijerph18189683/s1, File S1: Acción Conjunta Intergeneracional (face to face scales). The entire online survey sent to the participants.
