# Supplementary material for: Evaluation of the Benefits, Satisfaction, and Limitations of Intergenerational Face-to-Face Activities: A General Population Survey in Spain
Source: Int J Environ Res Public Health. 2021 Sep 14;18(18):9683. doi: 10.3390/ijerph18189683 (PMC8468254; doi:10.3390/ijerph18189683)
Supplement: Supplementary file 1 [file ijerph-18-09683-s001.zip › ijerph-1336674-supplementary.pdf]

Acción Conjunta Intergeneracional (ACIG)

PRESENTACIÓN Y CONFIDENCIALIDAD

PRESENTACIÓN

Se trata de una investigación cuyo objetivo es estudiar las **ACTIVIDADES CONJUNTAS REALIZADAS POR MIEMBROS DE DISTINTAS GENERACIONES**, para el diseño de pautas precisas de intervención que mejoren su bienestar y calidad de vida.

Para ello solicitamos su valiosa colaboración en la siguiente encuesta.

No existen respuestas correctas o incorrectas, sólo situaciones habituales diferentes. La mejor respuesta es la que refleje honestamente su realidad y punto de vista.

COMPROMISO DE CONFIDENCIALIDAD Y ANONIMATO

Sus respuestas son totalmente confidenciales y sólo tratadas a efectos estadísticos globales, de acuerdo con las normas éticas y deontológicas aplicables a toda investigación científica.

Esta encuesta es anónima, por lo que en ningún caso será posible su identificación a través de los datos que proporcione.

AGRADECIMIENTO

Le agradecemos su generosa y necesaria colaboración sin la cual no sería posible realizar esta investigación.

RECONOCIMIENTO

Se trata de una investigación pre-doctoral financiada por la JCyL (EDU/1083/2013) y la Unión Europea (FEDER 2014-2020) a A. Canedo, dentro del Grupo de Investigación de Excelencia (JCyL, GREX259, Dir JN García) cuyo objetivo es estudiar las **ACTIVIDADES CONJUNTAS REALIZADAS POR MIEMBROS DE DISTINTAS GENERACIONES**, para el diseño de pautas precisas de intervención que mejoren el bienestar de los participantes.

Acción Conjunta Intergeneracional (ACIG)

CONSENTIMIENTO INFORMADO

Si acepta colaborar en esta investigación, marque la opción "Acepto colaborar voluntariamente en el estudio" y complete la encuesta confidencial y anónima.

Para avanzar pulse **SIGUIENTE.**

En todo momento puede retroceder en la encuesta y modificar sus respuestas. Para ello, debe pulsar **ANTERIOR**.

Cuando haya concluido sus respuestas, pulse **FINALIZAR Y ENVIAR**.

1. ACEPTACIÓN DE COLABORACIÓN

- ○ Acepto colaborar voluntariamente en el estudio
- ○ No acepto colaborar en el estudio

universidad de león

Acción Conjunta Intergeneracional (ACIG)

DATOS DEMOGRÁFICOS (DDM)

2. Indique la fecha de nacimiento de su

	DD		MM		AAAA
MADRE		/		/	

3. Indique la fecha de nacimiento de su

	DD		MM		AAAA
PADRE		/		/	

4. Género

- ○ Hombre
- ○ Mujer

5. Fecha de nacimiento

	DD		MM		AAAA
Día / Mes / Año		/		/	

6. Edad

7. Nacionalidad

- Española
- Extranjera
- Española y otra

8. Lugar de procedencia

- Zona rural pueblo pequeño
- Zona rural pueblo grande
- Zona urbana ciudad pequeña
- Zona urbana ciudad grande

9. Núcleo poblacional de residencia

- Localidad de menos de 250 habitantes
- Localidad de 250-2000 habitantes
- Localidad de 2001-50.000 habitantes
- Localidad de 50.001-300.000 habitantes
- Localidad de más de 300.000 habitantes

10. Estado Civil

- No procede
- Soltero/a
- Casado/a o en pareja
- Viudo/a
- Separado/a
- Divorciado/a

11. ¿Dónde vive actualmente?

- En mi casa
- En casa de un familiar
- En casa de un amigo/a
- En un centro o residencia

12. ¿Con quién vive actualmente?

- Solo
- En pareja
- En pareja con hijos
- En pareja con nietos
- En pareja con hijos y nietos
- Con mis hijos
- Con mis nietos
- Con hijos y nietos
- Con mis padres
- Con mis abuelos
- Con mis padres y abuelos
- Con otros familiares
- Con amigos
- Otro (especifique)

13. ¿Qué nivel de estudios ha alcanzado?

- Estudios Primarios
- Estudios Secundarios
- Estudios Profesionales
- Estudios Universitarios

14. Autonomía Personal

	Solo/a	Con ayuda de un familiar/es	Con ayuda de un profesional/es	Con ayuda de otra persona/as
Habitualmente realizo las actividades de la vida diaria				

universidad de león

Acción Conjunta Intergeneracional (ACIG)

Actividad Laboral (DDM)

15. En relación con la actividad laboral, ¿en qué situación se encuentra?

- En desempleo
- Trabajando
- Percibiendo una pensión (contributiva o no contributiva)

Acción Conjunta Intergeneracional (ACIG)

Desempleo (DDM)

16. En relación a su situación de desempleo, se encuentra:

- ◯ Percibiendo la prestación por desempleo
- ◯ Cobrando la ayuda familiar
- ◯ No percibo ingresos fijos
- ◯ Otro (especifique)

Acción Conjunta Intergeneracional (ACIG)

Pensión (DDM)

17. Percibo una pensión por:

- ◯ Jubilación (ordinaria, anticipada, parcial, flexible, especial)
- ◯ Incapacidad permanente (total, absoluta o gran invalidez)
- ◯ Fallecimiento (viudedad, orfandad o en favor de familiares)
- ◯ Invalidez

Acción Conjunta Intergeneracional (ACIG)

Ocupación (DDM)

18. ¿Cuál es su ocupación actual o lo era antes del desempleo o la jubilación?

- Educación
- Sanidad
- Administración y Servicios
- Industria y Construcción
- Alimentación y Medio Natural
- Estudiante
- Otro (especifique)

19. Ejerce o ejercía dicha ocupación como:

- Empleador
- Empresario sin asalariados o trabajador independiente
- Miembro de una cooperativa
- Asalariado del sector público
- Asalariado del sector privado
- No compete

universidad de león

Acción Conjunta Intergeneracional (ACIG)

Ingresos (DDM)

20. Aproximadamente, ¿qué ingresos recibe al mes?

- Más de 2500 euros/mes
- Entre 2001-2500 euros/mes
- Entre 1501-2000 euros/mes
- Entre 1001-1500 euros/mes
- Ente 501-1000 euros/mes
- Menos de 500 euros/mes

universidad de león

Acción Conjunta Intergeneracional (ACIG)

Asociación (DDM)

21. ¿Pertenece a alguna asociación de afectados?

- ◯ Sí
- ◯ No

universidad de león

Acción Conjunta Intergeneracional (ACIG)

Tipo de Asociación (DDM)

22. Señale el tipo de asociación de afectados a la que pertenece:

- ☐ Discapacidad visual
- ☐ Discapacidad auditiva
- ☐ Discapacidad psíquica
- ☐ Discapacidad motórica
- ☐ Trastornos de aprendizaje
- ☐ Trastornos de conducta
- ☐ Trastornos de la comunicación
- ☐ Trastornos del espectro autista (TEA)
- ☐ Trastorno por déficit de atención con hiperactividad (TDAH)
- ☐ Otro (especifique)

universidad de león

Acción Conjunta Intergeneracional (ACIG)

APOYO SOCIAL PERCIBIDO (ASP)

Apoyo Instrumental (ASP)

23. Indique si recibe algún tipo de APOYO INSTRUMENTAL relacionado con su cuidado personal (aseo, seguimiento de dietas), desplazamiento y/o movilidad, la realización de las tareas del hogar, trámites y/o gestiones administrativas...

- ◯ Sí
- ◯ No

universidad de león

Acción Conjunta Intergeneracional (ACIG)

QUIÉNES - Apoyo Instrumental (ASP)

24. Señale por parte de QUIÉNES recibe dicho apoyo instrumental y el grado de satisfacción que ello le produce

	Nada satisfecho	Poco satisfecho	Algo satisfecho	Bastante satisfecho	Muy satisfecho
Mi pareja	◯	◯	◯	◯	◯
Mis hijos	◯	◯	◯	◯	◯
Mis nietos	◯	◯	◯	◯	◯
Mis padres	◯	◯	◯	◯	◯
Mis abuelos	◯	◯	◯	◯	◯
Mis hermanos	◯	◯	◯	◯	◯
Otros miembros de mi familia	◯	◯	◯	◯	◯
Mis amistades	◯	◯	◯	◯	◯
Mis vecinos, conocidos, compañeros de trabajo	◯	◯	◯	◯	◯
Asociaciones, organizaciones o personas en mi misma situación	◯	◯	◯	◯	◯
Servicios de salud, sociales y sus profesionales	◯	◯	◯	◯	◯
La escuela y sus profesionales	◯	◯	◯	◯	◯
Instituciones religiosas y sus miembros	◯	◯	◯	◯	◯

universidad de león

Acción Conjunta Intergeneracional (ACIG)

APOYO SOCIAL PERCIBIDO (ASP)

Apoyo Emocional (ASP)

25. Indique si recibe algún tipo de APOYO EMOCIONAL expresado por la vía del cariño, la confianza, la escucha, la empatía, la transmisión física de afectos, el acompañamiento, sentimientos de complicidad, de preocupación por su vida personal y/o familiar...

- Sí
- No

universidad de león

Acción Conjunta Intergeneracional (ACIG)

QUIÉNES - Apoyo Emocional (ASP)

26. Señale por parte de QUIÉNES recibe dicho apoyo emocional y el grado de satisfacción que ello le produce

	Nada satisfecho	Poco satisfecho	Algo satisfecho	Bastante satisfecho	Muy satisfecho
Mi pareja	○	○	○	○	○
Mis hijos	○	○	○	○	○
Mis nietos	○	○	○	○	○
Mis padres	○	○	○	○	○
Mis abuelos	○	○	○	○	○
Mis hermanos	○	○	○	○	○
Otros miembros de mi familia	○	○	○	○	○
Mis amistades	○	○	○	○	○
Mis vecinos, conocidos, compañeros de trabajo	○	○	○	○	○
Asociaciones, organizaciones o personas en mi misma situación	○	○	○	○	○
Servicios de salud, sociales y sus profesionales	○	○	○	○	○
La escuela y sus profesionales	○	○	○	○	○
Instituciones religiosas y sus miembros	○	○	○	○	○

Acción Conjunta Intergeneracional (ACIG)

ACTIVIDADES INTERGENERACIONALES (AIG)

ACTIVIDADES DE LA VIDA DIARIA Y DEL CUIDADO DE LA SALUD (AIG-VD)

27. ¿Realiza ACTIVIDADES DE LA VIDA DIARIA Y DEL CUIDADO DE LA SALUD con personas de otra generación relacionadas con el aseo, el seguimiento de dietas, la movilidad, los desplazamientos, la adquisición de bienes y servicios, la realización de las labores del hogar, trámites y/o gestiones administrativas, el cumplimiento de prescripciones médicas...de forma conjunta?

- Sí
- No

Acción Conjunta Intergeneracional (ACIG)

Actividades Vida Diaria y Cuidado Salud

28. Realizar actividades de la vida diaria junto con personas de otra generación produce BENEFICIOS para su:

	Totalmente en desacuerdo	En desacuerdo	Más bien en desacuerdo	Ni de acuerdo, ni en desacuerdo	Más bien de acuerdo	De acuerdo	Totalmente de acuerdo
Salud física							
Salud mental							
Estado de ánimo							
Relaciones interpersonales							
Autodeterminación o capacidad para decidir por sí mismo							
Implicación y participación social							
Bienestar material u económico							
Bienestar laboral o profesional							
Educación o formación académica							

29. ¿Con QUIÉNES realiza actividades de la vida diaria y del cuidado de la salud?

	Alguna vez al año	Alguna vez al mes	Alguna vez a la semana	Todos o casi todos los dias
Mi pareja				
Mi hijo/a				
Mi nieto/a				
Mi padre/madre				
Mi abuelo/a				
Mi hermano/a				
Otro miembro de mi familia				
Un amigo/a				
Un vecino/a, conocido/a				
Un colega o compañero/a de trabajo				
Una persona en mi misma situación (problemática)				
Persona / profesional de una asociación /organización / institución				
Persona / profesional de los servicios de salud, sociales o educativos				

30. EDAD de las personas con las que realiza actividades de la vida diaria y del cuidado de la salud:

	Menor de 6 años	6-14	15-20	21-39	40-59	60-65	66-70	71-75	Mayor de 75
Mi pareja									
Mi hijo/a									
Mi nieto/a									
Mi padre/madre									
Mi abuelo/a									
Mi hermano/a									
Otro miembro de mi familia									
Un amigo/a									
Un vecino/a, conocido/a									
Un colega o compañero/a de trabajo									
Una persona en mi misma situación (problemática)									
Persona / profesional de una asociación /organización / institución									
Persona / Profesional de los servicios de salud, sociales o educativos									

31. GÉNERO de las personas con las que realiza actividades de la vida diaria y del cuidado de la salud:

	Hombre	Mujer
Mi pareja	○	○
Mi hijo/a	○	○
Mi nieto/a	○	○
Mi padre/madre	○	○
Mi abuelo/a	○	○
Mi hermano/a	○	○
Otro miembro de mi familia	○	○
Un amigo/a	○	○
Un vecino/a, conocido/a	○	○
Un colega o compañero/a de trabajo	○	○
Una persona en mi misma situación (problemática)	○	○
Persona / profesional de una asociación /organización / institución	○	○
Persona / Profesional de los servicios de salud, sociales o educativos	○	○

32. AUTONOMÍA de las personas con las que realiza actividades de la vida diaria y del cuidado de la salud:

	No necesita ayuda	Necesita ayuda de un familiar/es	Necesita ayuda de un profesional/es	Necesita ayuda de otra persona/as
Mi pareja	○	○	○	○
Mi hijo/a	○	○	○	○
Mi nieto/a	○	○	○	○
Mi padre/madre	○	○	○	○
Mi abuelo/a	○	○	○	○
Mi hermano/a	○	○	○	○
Otro miembro de mi familia	○	○	○	○
Un amigo/a	○	○	○	○
Un vecino/a, conocido/a	○	○	○	○
Un colega o compañero/a de trabajo	○	○	○	○
Una persona en mi misma situación (problemática)	○	○	○	○
Persona / profesional de una asociación /organización / institución	○	○	○	○
Persona / Profesional de los servicios de salud, sociales o educativos	○	○	○	○

33. LIMITACIÓN de las personas con las que realiza actividades de la vida diaria y del cuidado de la salud:

	Otra	Sin discapacidad o dificultad de aprendizaje	Discapacidad visual	Discapacidad auditiva	Discapacidad psíquica	Discapacidad motórica	Trastorno de aprendizaje	Trastorno de conducta	Trastorno de comunicación	Trastorno del espectro autista (TEA)	Trastorno de déficit de atención con hiperactividad (TDAH)
Mi pareja	◯	◯	◯	◯	◯	◯	◯	◯	◯	◯	◯
Mi hijo/a	◯	◯	◯	◯	◯	◯	◯	◯	◯	◯	◯
Mi nieto/a	◯	◯	◯	◯	◯	◯	◯	◯	◯	◯	◯
Mi padre/madre	◯	◯	◯	◯	◯	◯	◯	◯	◯	◯	◯
Mi abuelo/a	◯	◯	◯	◯	◯	◯	◯	◯	◯	◯	◯
Mi hermano/a	◯	◯	◯	◯	◯	◯	◯	◯	◯	◯	◯
Otro miembro de mi familia	◯	◯	◯	◯	◯	◯	◯	◯	◯	◯	◯
Un amigo/a	◯	◯	◯	◯	◯	◯	◯	◯	◯	◯	◯
Un vecino/a, conocido/a	◯	◯	◯	◯	◯	◯	◯	◯	◯	◯	◯
Un colega o compañero/a de trabajo	◯	◯	◯	◯	◯	◯	◯	◯	◯	◯	◯
Una persona en mi misma situación (problemática)	◯	◯	◯	◯	◯	◯	◯	◯	◯	◯	◯
Persona / profesional de una asociación /organización / institución	◯	◯	◯	◯	◯	◯	◯	◯	◯	◯	◯
Persona / Profesional de los servicios de salud, sociales o educativos	◯	◯	◯	◯	◯	◯	◯	◯	◯	◯	◯

34. SATISFACCIÓN que le produce realizar junto con estas personas actividades de la vida diaria y del cuidado de la salud:

	Nada satisfecho	Poco satisfecho	Algo satisfecho	Bastante satisfecho	Muy satisfecho
Mi pareja	○	○	○	○	○
Mi hijo/a	○	○	○	○	○
Mi nieto/a	○	○	○	○	○
Mi padre/madre	○	○	○	○	○
Mi abuelo/a	○	○	○	○	○
Mi hermano/a	○	○	○	○	○
Otro miembro de mi familia	○	○	○	○	○
Un amigo/a	○	○	○	○	○
Un vecino/a, conocido/a	○	○	○	○	○
Un colega o compañero/a de trabajo	○	○	○	○	○
Una persona en mi misma situación (problemática)	○	○	○	○	○
Un profesional de una asociación u organización	○	○	○	○	○
Un profesional de los servicios de salud, sociales u educativos	○	○	○	○	○

universidad de león

Acción Conjunta Intergeneracional (ACIG)

ACTIVIDADES FORMATIVAS, CULTURALES Y DE OCIO (AIG-FORM)

35. ¿Realiza ACTIVIDADES FORMATIVAS, CULTURALES y de OCIO con personas de otra generación, relacionadas con el aprendizaje o la enseñanza de la lectura y la escritura, ayudar o recibir apoyo en los estudios, la asistencia a clases de música o idiomas, a cursos de dibujo o pintura, talleres de bricolaje o jardinería, conferencias, cine, teatro... de forma conjunta?

○ Sí

○ No

universidad de león

Actividades Formativas, Culturales y de Ocio

36. Realizar actividades formativas, culturales y de ocio junto con personas de otra generación produce BENEFICIOS para su:

	Totalmente en desacuerdo	En desacuerdo	Más bien en desacuerdo	Ni de acuerdo, ni en desacuerdo	Más bien de acuerdo	De acuerdo	Totalmente de acuerdo
Salud física	○	○	○	○	○	○	○
Salud mental	○	○	○	○	○	○	○
Estado de ánimo	○	○	○	○	○	○	○
Relaciones interpersonales	○	○	○	○	○	○	○
Autodeterminación o capacidad para decidir por sí mismo	○	○	○	○	○	○	○
Implicación y participación social	○	○	○	○	○	○	○
Bienestar material u económico	○	○	○	○	○	○	○
Bienestar laboral o profesional	○	○	○	○	○	○	○
Educación o formación académica	○	○	○	○	○	○	○

37. ¿Con QUIÉNES realiza actividades formativas, culturales y de ocio?

	Alguna vez al año	Alguna vez al mes	Alguna vez a la semana	Todos o casi todos los dias
Mi pareja	○	○	○	○
Mi hijo/a	○	○	○	○
Mi nieto/a	○	○	○	○
Mi padre/madre	○	○	○	○
Mi abuelo/a	○	○	○	○
Mi hermano/a	○	○	○	○
Otro miembro de mi familia	○	○	○	○
Un amigo/a	○	○	○	○
Un vecino/a, conocido/a	○	○	○	○
Un colega o compañero/a de trabajo	○	○	○	○
Una persona en mi misma situación (problemática)	○	○	○	○
Un profesional de una asociación u organización	○	○	○	○
Un profesional de los servicios de salud, sociales u educativos	○	○	○	○

38. EDAD de las personas con las que realiza actividades formativas, culturales y de ocio:

	Menor de 6 años	6-14	15-20	21-39	40-59	60-65	66-70	71-75	Mayor de 75
Mi pareja									
Mi hijo/a									
Mi nieto/a									
Mi padre/madre									
Mi abuelo/a									
Mi hermano/a									
Otro miembro de mi familia									
Un amigo/a									
Un vecino/a, conocido/a									
Un colega o compañero/a de trabajo									
Una persona en mi misma situación (problemática)									
Persona / profesional de una asociación /organización / institución									
Persona / Profesional de los servicios de salud, sociales o educativos									

39. GÉNERO de las personas con las que realiza actividades formativas, culturales y de ocio:

	Hombre	Mujer
Mi pareja		
Mi hijo/a		
Mi nieto/a		
Mi padre/madre		
Mi abuelo/a		
Mi hermano/a		
Otro miembro de mi familia		
Un amigo/a		
Un vecino/a, conocido/a		
Un colega o compañero/a de trabajo		
Una persona en mi misma situación (problemática)		
Persona / profesional de una asociación /organización / institución		
Persona / Profesional de los servicios de salud, sociales o educativos		

40. AUTONOMÍA de las personas con las que realiza actividades formativas, culturales y de ocio:

	No necesita ayuda	Necesita ayuda de un familiar/es	Necesita ayuda de un profesional/es	Necesita ayuda de otra persona/as
Mi pareja	○	○	○	○
Mi hijo/a	○	○	○	○
Mi nieto/a	○	○	○	○
Mi padre/madre	○	○	○	○
Mi abuelo/a	○	○	○	○
Mi hermano/a	○	○	○	○
Otro miembro de mi familia	○	○	○	○
Un amigo/a	○	○	○	○
Un vecino/a, conocido/a	○	○	○	○
Un colega o compañero/a de trabajo	○	○	○	○
Una persona en mi misma situación (problemática)	○	○	○	○
Persona / profesional de una asociación /organización / institución	○	○	○	○
Persona / Profesional de los servicios de salud, sociales o educativos	○	○	○	○

41. LIMITACIÓN de las personas con las que realiza actividades formativas, culturales y de ocio:

	Otra	Sin discapacidad o dificultad de aprendizaje	Discapacidad visual	Discapacidad auditiva	Discapacidad psíquica	Discapacidad motórica	Trastorno de aprendizaje	Trastorno de conducta	Trastorno de comunicación	Trastorno del espectro autista (TEA)	Trastorno de déficit de atención con hiperactividad (TDAH)
Mi pareja											
Mi hijo/a											
Mi nieto/a											
Mi padre/madre											
Mi abuelo/a											
Mi hermano/a											
Otro miembro de mi familia											
Un amigo/a											
Un vecino/a, conocido/a											
Un colega o compañero/a de trabajo											
Una persona en mi misma situación (problemática)											
Persona / profesional de una asociación /organización / institución											
Persona / Profesional de los servicios de salud, sociales o educativos											

42. SATISFACCIÓN que le produce realizar junto con estas personas actividades formativas, culturales y de ocio:

	Nada satisfecho	Poco satisfecho	Algo satisfecho	Bastante satisfecho	Muy satisfecho
Mi pareja	○	○	○	○	○
Mi hijo/a	○	○	○	○	○
Mi nieto/a	○	○	○	○	○
Mi padre/madre	○	○	○	○	○
Mi abuelo/a	○	○	○	○	○
Mi hermano/a	○	○	○	○	○
Otro miembro de mi familia	○	○	○	○	○
Un amigo/a	○	○	○	○	○
Un vecino/a, conocido/a	○	○	○	○	○
Un colega o compañero/a de trabajo	○	○	○	○	○
Una persona en mi misma situación (problemática)	○	○	○	○	○
Persona / profesional de una asociación /organización / institución	○	○	○	○	○
Persona / Profesional de los servicios de salud, sociales o educativos	○	○	○	○	○

universidad de león

Acción Conjunta Intergeneracional (ACIG)

ACTIVIDADES FÍSICAS Y DEPORTIVAS (AIG-FYD)

43. ¿Realiza algún tipo de ACTIVIDAD FÍSICA O PRACTICA DEPORTIVA tales como subir escaleras, salir a pasear, a correr, jugar a la petanca, al fútbol, etc., junto con personas de otra generación?

○ Sí

○ No

universidad de león

Acción Conjunta Intergeneracional (ACIG)

Actividades Físicas y Deportivas

44. Realizar actividades físicas y deportivas junto con personas de otra generación produce BENEFICIOS para su:

	Totalmente en desacuerdo	En desacuerdo	Más bien en desacuerdo	Ni de acuerdo, ni en desacuerdo	Más bien de acuerdo	De acuerdo	Totalmente de acuerdo
Salud física	○	○	○	○	○	○	○
Salud mental	○	○	○	○	○	○	○
Estado de ánimo	○	○	○	○	○	○	○
Relaciones interpersonales	○	○	○	○	○	○	○
Autodeterminación o capacidad para decidir por sí mismo	○	○	○	○	○	○	○
Implicación y participación social	○	○	○	○	○	○	○
Bienestar material u económico	○	○	○	○	○	○	○
Bienestar laboral o profesional	○	○	○	○	○	○	○
Educación o formación académica	○	○	○	○	○	○	○

45. ¿Con QUIÉNES realiza actividades físicas y deportivas?

	Alguna vez al año	Alguna vez al mes	Alguna vez a la semana	Todos o casi todos los dias
Mi pareja	○	○	○	○
Mi hijo/a	○	○	○	○
Mi nieto/a	○	○	○	○
Mi padre/madre	○	○	○	○
Mi abuelo/a	○	○	○	○
Mi hermano/a	○	○	○	○
Otro miembro de mi familia	○	○	○	○
Un amigo/a	○	○	○	○
Un vecino/a, conocido/a	○	○	○	○
Un colega o compañero/a de trabajo	○	○	○	○
Una persona en mi misma situación (problemática)	○	○	○	○
Persona / profesional de una asociación /organización / institución	○	○	○	○
Persona / Profesional de los servicios de salud, sociales o educativos	○	○	○	○

46. EDAD de las personas con las que realiza actividades físicas y deportivas:

	Menor de 6 años	6-14	15-20	21-39	40-59	60-65	66-70	71-75	Mayor de 75
Mi pareja									
Mi hijo/a									
Mi nieto/a									
Mi padre/madre									
Mi abuelo/a									
Mi hermano/a									
Otro miembro de mi familia									
Un amigo/a									
Un vecino/a, conocido/a									
Un colega o compañero/a de trabajo									
Una persona en mi misma situación (problemática)									
Persona / profesional de una asociación /organización / institución									
Persona / Profesional de los servicios de salud, sociales o educativos									

47. GÉNERO de las personas con las que realiza actividades físicas y deportivas:

	Hombre	Mujer
Mi pareja		
Mi hijo/a		
Mi nieto/a		
Mi padre/madre		
Mi abuelo/a		
Mi hermano/a		
Otro miembro de mi familia		
Un amigo/a		
Un vecino/a, conocido/a		
Un colega o compañero/a de trabajo		
Una persona en mi misma situación (problemática)		
Un profesional de una asociación u organización		
Un profesional de los servicios de salud, sociales u educativos		

48. AUTONOMÍA de las personas con las que realiza actividades físicas y deportivas:

	No necesita ayuda	Necesita ayuda de un familiar/es	Necesita ayuda de un profesional/es	Necesita ayuda de otra persona/as
Mi pareja	◯	◯	◯	◯
Mi hijo/a	◯	◯	◯	◯
Mi nieto/a	◯	◯	◯	◯
Mi padre/madre	◯	◯	◯	◯
Mi abuelo/a	◯	◯	◯	◯
Mi hermano/a	◯	◯	◯	◯
Otro miembro de mi familia	◯	◯	◯	◯
Un amigo/a	◯	◯	◯	◯
Un vecino/a, conocido/a	◯	◯	◯	◯
Un colega o compañero/a de trabajo	◯	◯	◯	◯
Una persona en mi misma situación (problemática)	◯	◯	◯	◯
Persona / profesional de una asociación /organización / institución	◯	◯	◯	◯
Persona / Profesional de los servicios de salud, sociales o educativos	◯	◯	◯	◯

49. LIMITACIÓN de las personas con las que realiza actividades físicas y deportivas:

	Otra	Sin discapacidad o dificultad de aprendizaje	Discapacidad visual	Discapacidad auditiva	Discapacidad psíquica	Discapacidad motórica	Trastorno de aprendizaje	Trastorno de conducta	Trastorno de comunicación	Trastorno del espectro autista (TEA)	Trastorno de déficit de atención con hiperactividad (TDAH)
Mi pareja	○	○	○	○	○	○	○	○	○	○	○
Mi hijo/a	○	○	○	○	○	○	○	○	○	○	○
Mi nieto/a	○	○	○	○	○	○	○	○	○	○	○
Mi padre/madre	○	○	○	○	○	○	○	○	○	○	○
Mi abuelo/a	○	○	○	○	○	○	○	○	○	○	○
Mi hermano/a	○	○	○	○	○	○	○	○	○	○	○
Otro miembro de mi familia	○	○	○	○	○	○	○	○	○	○	○
Un amigo/a	○	○	○	○	○	○	○	○	○	○	○
Un vecino/a, conocido/a	○	○	○	○	○	○	○	○	○	○	○
Un colega o compañero/a de trabajo	○	○	○	○	○	○	○	○	○	○	○
Una persona en mi misma situación (problemática)	○	○	○	○	○	○	○	○	○	○	○
Persona / profesional de una asociación /organización / institución	○	○	○	○	○	○	○	○	○	○	○
Persona / Profesional de los servicios de salud, sociales o educativos	○	○	○	○	○	○	○	○	○	○	○

50. SATISFACCIÓN que le produce realizar junto con estas personas actividades físicas y deportivas:

	Nada satisfecho	Poco satisfecho	Algo satisfecho	Bastante satisfecho	Muy satisfecho
Mi pareja	○	○	○	○	○
Mi hijo/a	○	○	○	○	○
Mi nieto/a	○	○	○	○	○
Mi padre/madre	○	○	○	○	○
Mi abuelo/a	○	○	○	○	○
Mi hermano/a	○	○	○	○	○
Otro miembro de mi familia	○	○	○	○	○
Un amigo/a	○	○	○	○	○
Un vecino/a, conocido/a	○	○	○	○	○
Un colega o compañero/a de trabajo	○	○	○	○	○
Una persona en mi misma situación (problemática)	○	○	○	○	○
Persona / profesional de una asociación /organización / institución	○	○	○	○	○
Persona / Profesional de los servicios de salud, sociales o educativos	○	○	○	○	○

universidad de león

Acción Conjunta Intergeneracional (ACIG)

ACTIVIDADES INTERGENERACIONALES VIRTUALES (AIGV)

ACTIVIDADES CON HERRAMIENTAS DE COMUNICACIÓN VIRTUALES (AIGV-COM)

51. ¿Utiliza HERRAMIENTAS DE COMUNICACIÓN VIRTUALES (Skype, Whatsapp, Correo electrónico, etc.) con personas de otra generación de forma conjunta?

○ Sí

○ No

universidad de león
